# The release of IL-31 and IL-13 after nasal allergen challenge and their relation to nasal symptoms

**DOI:** 10.1186/2045-7022-2-13

**Published:** 2012-08-01

**Authors:** Ralf Baumann, Matthaeus Rabaszowski, Igor Stenin, Maria Gaertner-Akerboom, Kathrin Scheckenbach, Jens Wiltfang, Joerg Schipper, Martin Wagenmann

**Affiliations:** 1 Department of Otorhinolaryngology, Head and Neck Surgery, Heinrich-Heine-University Duesseldorf, Duesseldorf, Germany; 2 Laboratory of Molecular Neurobiology, Clinics for Psychiatry und Psychotherapy, University of Duisburg-Essen, Essen, Germany

**Keywords:** Nasal allergen, Nasal secretion, IL-13, IL-31, Kinetics

## Abstract

**Background:**

IL-31, a recently discovered member of the gp130/IL-6 cytokine family, is mainly expressed by human mast cells and T helper type 2 cells. IL-31 is a key trigger of atopic dermatitis. Recent studies also suggest a role of IL-31 in the pathogenesis of other allergic diseases including allergic rhinitis. In the present study we studied the release of IL-31 and IL-13 in allergen-challenged allergic rhinitis patients.

**Methods:**

Seven seasonal allergic volunteers underwent unilateral nasal provocation with allergen (and a control challenge) with the disc method out of the allergy season. Nasal symptom scores (rhinorrhea, itching, sneezing, obstruction) and bilateral nasal secretions were quantified before and after allergen provocation. IL-13 and IL-31 in nasal secretions and serum were measured by electrochemiluminescent immunoassay or ELISA, respectively.

**Results:**

Nasal allergen challenge induced the typical clinical symptoms and physiological changes. IL-31 and IL-13 in nasal secretions increased in four and five, respectively, volunteers at 5 h after allergen but not after control challenge. We observed correlation trends between nasal IL-31 concentrations and IL-13 concentrations (r = 0.9, p = 0.002), and IL-31 contents and symptom scores (r = 0.9, p = 0.013) 5 h after allergen provocation. No IL-31 could be detected contralaterally or systemically in the sera.

**Conclusions:**

The observed local upregulation of IL-31 mainly during the late phase reaction after nasal allergen challenge suggests a role of IL-31 in allergic rhinitis. In which way IL-31 modulates the inflammatory reaction and type 2 responses in allergic rhinitis remains to be investigated.

## Background

IL-31 is a recently discovered member of the gp130/IL-6 cytokine family, which includes IL-6, IL-11, IL-27, oncostatin M (OSM), leukemia inhibitory factor (LIF), ciliary neurotrophic factor, neuropoietin, cardiotrophin-1, and cardiotrophin-like cytokine [1,2]. IL-31 signals through a heterodimeric receptor composed of the IL-31 receptor alpha (IL-31RA) and the OSM receptor beta (OSMR). IL-31RA has been identified as a gp130-like receptor showing 28% homology to gp130, the common signalling receptor subunit of the family of IL-6-type cytokines [3-5].

IL-31 is expressed by human mast cells [6] and by CD4^+^ T cells, particularly activated T helper type 2 (TH2) cells [1], and skin-homing CD45RO^+^ cutaneous lymphocyte-associated antigen-positive T cells [7]. IL-31 receptors are expressed on a broad spectrum of immune and non-immune cells including activated monocytes, macrophages, eosinophils, basophils, dorsal root ganglia, keratinocytes, and epithelial cells [2,8,9].

Several studies support a role for IL-31 in atopic dermatitis and other epithelial pathologies. Mice treated with intradermal injection of IL-31 or transgenic mice overexpressing IL-31 presented increased scratching behavior and developed severe dermatitis [1]. A neutralizing anti-IL-31 monoclonal antibody ameliorated scratching behavior in a mouse model of dermatitis [10]. A common haplotype of the IL-31 gene was characterized as a risk haplotype for nonatopic eczema [11]. IL-31 mRNA was reported to be overexpressed in pruritic atopic dermatitis but not in psoriasis which is a mainly TH1-dependent inflammatory disease, confirming an involvement of IL-31 in TH2-mediated skin diseases [2]. In addition, IL-31 mRNA expression was also increased in skin samples of patients with allergic contact dermatitis and was correlated with IL-4 and IL-13 levels [12]. Increased levels of IL-31 protein in serum and mRNA in peripheral blood mononuclear cells (PBMCs) were found in patients with allergic asthma [13]. Very recent evidence showed pollen antigen-induced IL-31 protein production from PBMCs in patients with allergic rhinitis in association with the severity of allergic rhinitis [14]. This IL-31 production was significantly and positively correlated with the production of IL-5 and IL-13 [14].

IL-13, which is a central type 2 cytokine [15], and its receptors have been shown to be produced by most immune cells [5,16]. IL-13 is involved in B-cell maturation, differentiation and IgE isotype switching. It down-modulates macrophage activity, thereby inhibiting the production of pro-inflammatory cytokines and chemokines [5]. Many lines of evidence support an important role of IL-13 in the pathophysiology of allergic asthma and allergic rhinitis [17-20].

Allergic rhinitis is the most frequent allergic disease and affects approximately 10% to 25% of the population [21-23]. It invariably involves inflammation of the nasal mucosa and often also the mucosa of other organs such as eyes and sinuses [23,24]. To further elucidate the potential role of IL-31 in allergic rhinitis, we studied the release of IL-31 and IL-13 into nasal secretions and serum after unilateral allergen challenge in seasonal allergic volunteers. Nasal allergen provocation allows to differentiate between the early and late phase of the allergic reaction in the nose and to describe the time course of release for cytokines and other mediators [25,26]. We used the filter paper disc method which allows the separate investigation of ipsi- and contralateral effects after an unilateral allergen challenge [25,27].

## Methods

### Subjects

Eleven healthy, non-smoking subjects with seasonal allergic rhinitis were included in the study. All subjects provided a written informed consent and the study was approved by the Ethics Committee of the Heinrich-Heine University. The subjects were selected by means of symptom history assessed by questionnaire and a skin prick test showing sensitization for seasonal grass (grass mix, Allergopharma Joachim Ganzer KG, Reinbek Germany) or tree (tree II mix (birch, euopean beech, plane, oak); Allergopharma) pollen allergens and negative results for common perennial allergens. The skin prick test was performed according to the guidelines of the German Society for Allergology and Clinical Immunology [28]. In brief, after cleaning the skin with alcohol the positions of allergens were marked by numbered strips on the volar sides of both forearms. A drop of each allergen was applied to the corresponding spot of the skin. Saline (negative control) and histamine dihydrochloride solution (positive control) (Allergopharma) were included in the test. The skin was gently pricked with a sterile lancet (Allergopharma) through the drops using a perpendicular motion. Between each prick the lancet was wiped on a gauze to prevent contamination of the following site. Eventually, the drops were blotted from the skin. The reaction was read after 15 to 20 min and the presence of a mean wheal diameter of at least 3 mm was regarded as a positive [28-30]. In case of positive reactions to both grass and tree pollen the allergen with the stronger reaction was chosen for nasal provocation. Throughout the study the subjects were out of allergy season to avoid natural allergen exposition. None of the volunteers took any medication except oral contraceptives or showed evidence of sinusitis or active rhinitis within months prior to challenge or received specific immunotherapy with allergens in the previous 3 years. Four subjects had to be excluded prior to analysis because of procedural adverse events (nasal bleedings; two excluded), incompliance (one excluded) or lack of positive symptom scores after nasal allergen challenge (one excluded). The remaining subjects were three men and four women with an age range of 19–53 years and a mean age of 31 years.

### Localized nasal challenge

The allergen challenge and collection of nasal secretions were carried out by a modified version of the filter disc method [25]. Seventy-five μl of allergen solution (Allergopharma) in a concentration of 50.000 BU/ml were pipetted on a filter disc (10 mm diameter, 1.2 mm thickness; Shandon filter cards; Shandon Inc., Pittsburgh, PA, USA). Each challenge disc therefore contained 3.750 BU of allergen. It was placed on the anterior portion of the nasal septum for 1 minute. Ten minutes, two, five, and 24 hours after challenge nasal secretions were collected by applying pre-weighed filter discs to the same spot of the septum for 45 seconds in the ipsilateral nostril and to the corresponding area of the contralateral nasal septum.

Symptoms were quantified by visual analog scales for secretion, obstruction and itching. Sneezes were counted and the nasal air flow was evaluated by anterior rhinomanometry (Atmos Rhinomanometer 300; ATMOS MedizinTechnik; Lenzkirch, Germany) at all measurement points. Prior to the challenge anatomic anomalies were ruled out by anterior rhinoscopy. To wash out preexisting mediators and acquire bilateral baseline nasal secretions, five consecutive nasal lavages were performed with 0.9% isotonic sodium chloride solution (B. Braun Melsungen A.G., Melsungen, Germany).

To determine potential unspecific reactions, a disc challenge with diluent alone (sterile saline; Allergopharma) was performed prior to the allergen challenge. Furthermore, control challenges using the diluent alone were performed (analog to the allergen challenge protocol) either at least four days before or at least two weeks after the allergen challenge.

After collection each filter disc were weighed, placed in a 2 ml Cryo S PP round bottom tube (Greiner Bio-One, Frickenhausen, Germany) and eluted in 500 μl of 0.9% sodium chloride solution for approximately 3 h while gently shaking at 4°C. After removal of the disk, the eluate was centrifuged at 2800 rpm (Biofuge 15R; Heraeus Instruments; Duesseldorf, Germany) to remove mucus and cell material. The supernatant was aliquoted into 0.5 ml tubes (LoBind; Eppendorf AG; Hamburg, Germany) and stored at −80°C until analysis.

### Serum

Five ml of peripheral blood were collected using SST II tubes (Becton, Dickinson and Company (BD), Heidelberg, Germany) from all participants before and 5 and 24 h after nasal allergen challenge. After clotting of the blood, serum was separated by centrifugation at 4°C and stored in aliquots at −80°C.

### IL-31 enzyme-linked immunosorbent assay

IL-31 levels in nasal secretions and in serum were analyzed using a commercially available pre-coated enzyme-linked-immunosorbent-assay (ELISA) (LEGEND MAX™ Human IL-31 ELISA Kit; BioLegend, Inc.; San Diego, CA, USA) with a sensitivity of <7.3 pg/ml. The IL-31 ELISA was performed according to the manufacturer’s instructions and the ELISA-plates were read on an Elisa Reader (DTX 880 Multimode Detector, Beckmann Coulter GmbH, Krefeld, Germany). Duplicate concentration values were ≤20%. The concentrations and contents of nasal IL-31 were calculated considering the amount of generated nasal secretions.

### Electrochemiluminescent IL-13 Immunoassay

IL-13 levels in nasal secretion samples were analyzed using a commercially available ultrasensitive pre-coated electrochemiluminescence immunoassay (Human IL-13 Ultra-Sensitive Kit; Meso Scale Discovery (MSD), Gaithersburg, MD, USA) with a sensitivity of <6.9 pg/ml. The IL-13 standard was diluted either in isotonic Sodium Chloride Solution/1% Blocker A (MSD) or Diluent 2 (MSD) for the investigation of nasal secretions or serum, respectively. All incubations of the immunoassay were done sealed on an orbital shaker (Eppendorf) at room temperature. Plates were blocked with 25 μl Diluent 2 (MSD) for 30 min, before 25 μl of zero standards in triplicates and of standards and samples in duplicates were added. After 2 ½ h of incubation and three washes, twenty-five μl of anti-IL-13 antibody labelled with Sulfo-Tag (MSD) were added to each well and incubated for additional 2 h protected from light. After further four washes, 150 μl of 2x read buffer (MSD) were added per well and the plate was immediately read on the Sector Imager 6000 (MSD). Raw data were analyzed using the Discovery Workbench 3.0 software (MSD). The concentrations and total amounts of nasal IL-13 were calculated considering the amount of generated nasal secretions.

### Statistics

Because the data were not normally distributed, nonparametric statistics were employed. Statistical analyses were performed using Medcalc software 12.1.0 (Kagi, Berkeley, CA). Graphics were done using GraphPad Prism (GraphPad, San Diego, CA, USA). To examine differences between points on either the diluent or the allergen challenge curves, Friedman analysis of variance (ANOVA) was used. If a significant overall difference was found, *post-hoc* analysis was performed using the method given by Conover [31] to identify time points showing significantly different levels compared to the baseline and diluent time points. Correction for multiple testing has not been applied. Correlations were evaluated by the Spearman rank test and Kendall´s tau test. P < 0.05 (two-tailed) was considered statistically significant. If not otherwise stated mean ± SEM is displayed.

## Results

**Figure 1:**
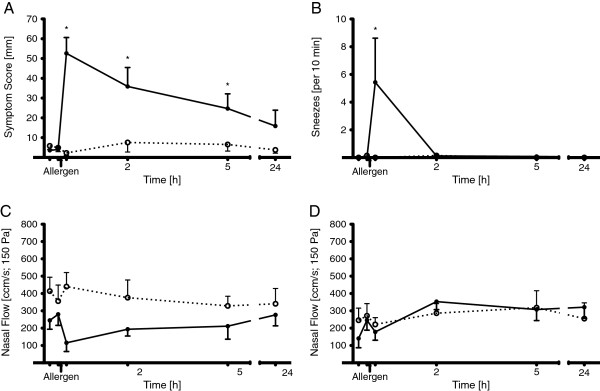
**Time course of symptoms and nasal airway flow after unilateral nasal allergen challenge.** Symptom scores (**A**) and sneezes (**B**) and ipsilateral (**C**) or contralateral (**D**) nasal airway flow after unilateral nasal allergen challenge are shown. Nasal air flow was evaluated by anterior rhinomanometry. Nasal symptoms and airway flow were evaluated before and at all time-points following allergen challenge. The dashed line represents values obtained after control challenge on a separate day using the same protocol. Values presented are means ± SEM (n = 7; *p < 0.05).

**Figure 2:**
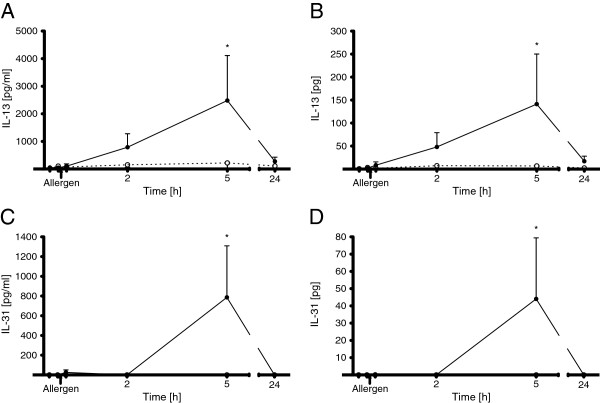
**Time course of IL-13 and IL-31 in ipsilateral nasal secretions after unilateral nasal allergen challenge.** The IL-13 (**A**, **B**) and IL-31 (**C**, **D**) concentrations (**A**, **C**) and contents (**B**, **D**) in ipsilateral nasal secretions are shown. Nasal secretions were collected before and at all time-points following allergen challenge. The dashed line represents values obtained after control challenge on a separate day using the same protocol. Values presented are means ± SEM (n = 7; *p < 0.05).

Following nasal allergen challenge, nasal symptoms and cytokine levels increased significantly (Figures 1 and 2). Postallergen symptom score (mean of itching, obstruction and secretion score) was elevated rapidly within 10 min [diluent: 2.3 ± 1.3 mm; allergen (10 min): 53 ± 8 mm] and gradually abated during the following 24 h, remaining significantly elevated until 5 h after allergen challenge [24.7 ± 7.5 mm] (Figure 1A). In contrast, control challenge with the diluent alone did not cause any statistically significant changes (Figure 1A). The number of sneezes was significantly increased in the minutes after the allergen challenge [diluent: 0 ± 0; allergen (10 min): 5.4 ± 3.2] returning soon to low levels and zero thereafter (Figure 1B). Nasal flow measured by rhinomanometry decreased from 280 ± 64 cm^3^/s (diluent) to 115 ± 51 cm^3^/s 10 min after allergen challenge and gradually returned to the original level until the 24 h measuring time point on the ipsilateral side (Figure 1C). On the contralateral side its drop immediately after the allergen provocation was less pronounced from 246 ± 150 cm^3^/s to 178 ± 128 cm^3^/s (Figure 1D).

IL-13 was found in very low amounts in nasal secretions before and 10 min after nasal provocation, but its concentration and content rose 2 h and more pronounced 5 h after provocation [concentration 2482 ± 1629 pg/ml; content 141 ± 108.9 pg] before falling again to low levels after 24 h (Figure 2A and B; Friedman ANOVA: p = 0.006 (A), p = 0.031 (B)). In one of the seven subjects, IL-13 was only detectable after 24 h. On the contralateral side, IL-13 levels remained very low (data not shown). No statistically significant changes for IL-13 concentration and content were observed after control challenge with the diluent alone (Figure 2A and B).

Generally, IL-31 was below the limit of detection of the assay in nasal secretions before nasal provocation. In four of the seven allergic volunteers nasal IL-31 was still undetectable at the early response but increased 5 h after provocation on the ipsilateral side [concentration 786 ± 522 pg/ml; content 44 ± 35.4 pg] before falling again to undetectable levels after 24 h (Figure 2C and D; Friedman ANOVA: p < 0.001 (C, D)). In a fifth subject a low nasal IL-31 level was detected exclusively at the 10 min time point. In the remaining two subjects, no nasal IL-31 was detected at any measuring time point after nasal provocation. On the contralateral side, no IL-31 was measured (data not shown). No IL-31 could be detected after control challenge (Figure 2C and D).

**Figure 3:**
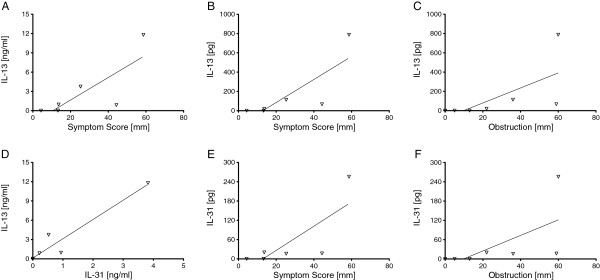
**Correlation trends of cytokine levels with nasal symptom scores and each other.A**, **B** and **E**: Symptom scores with IL-13 concentrations (**A**), IL-13 contents (**B**) and IL-31 contents (**E**); **C**, **F**: Obstruction with IL-13 contents (**C**) and IL-31 contents (**F**); **D**: IL-13 and IL-31 concentrations. Nasal symptoms were calculated as the mean of secretion, obstruction and itching (visual analog scale (VAS)). The corresponding correlation coefficients and p-values calculated by the Spearman rank test as well as the Kendall´s tau test are depicted in Tables 1 and 2 (n = 7).

Nasal concentrations and contents of IL-31 and IL-13 at 5 h were tested for correlations to each other as well as to symptom scores at 5 h (Tables 1 and 2). Due to the low number of study participants and some zero values of IL-31 and IL-13 levels at 5 h, Kendall´s tau test was performed besides the Spearman rank test (Tables 1 and 2). For the same reason, we speak of correlation trends instead of correlations also in case of significant p-values. In Figure 3, only those association trends with significant p-values according to the Kendall´s tau test are depicted. Nasal IL-13 concentrations and contents showed correlation trends with nasal symptom scores (concentration: Spearman rank test: r = 0.86, p = 0.012; Kendall’s tau test: tau = 0.75, p = 0.027; content: r = 0.94, p = 0.002; tau = 0.85, p = 0.012) (Figure 3A and B; Table 1 and 2). There was a correlation trend between symptom scores with IL-31 contents (r = 0.86, p = 0.013; tau = 0.74, p = 0.031) (Figure 3E; Table 2). IL-31 showed an association trend with IL-13 concentrations (r = 0.94, p = 0.002; tau = 0.84, p = 0.013) (Figure 3D; Table 1). When we compared nasal itching, obstruction, and secretion at 5 h with nasal concentrations and contents of IL-31 and IL-13 at 5 h using Kendall´s tau test (Tables 1 and 2), we found an association trend for obstruction at 5 h with both IL-31 and IL-13 contents (tau = 0.72, p = 0.035 and tau = 0.68, p = 0.045, respectively) (Figure 3C and F; Table 2).

**Table 1:** Associations between nasal cytokine concentrations and symptoms (n = 7)

	**IL-13 at 5 h**				**IL-31 at 5 h**			
	rho^*^	**(p)**	tau^**^	**(p)**	rho^*^	**(p)**	tau^**^	**(p)**
Symptom scores at 5 h	0.86	(0.012)	0.75	(0.027)	0.82	(0.023)	*0.63*	*(0.067)*
Obstruction at 5 h	0.77	(0.041)	*0.59*	*(0.091)*	0.82	(0.025)	*0.62*	*(0.074)*
IL-13 at 5 h	-				0.94	(0.002)	0.84	(0.013)

^*^ Spearman correlation analysis.

^**^ Kendall correlation analysis.

Non-significant correlations are in italic.

**Table 2:** Associations between nasal cytokine contents and symptoms (n = 7)

	**IL-13 at 5 h**				**IL-31 at 5 h**			
	rho^*^	**(p)**	tau^**^	**(p)**	rho^*^	**(p)**	tau^**^	**(p)**
Symptom scores at 5 h	0.94	(0.002)	0.85	(0.012)	0.86	(0.013)	0.74	(0.031)
Obstruction at 5 h	0.85	(0.016)	0.68	(0.045)	0.85	(0.015)	0.72	(0.035)
IL-13 at 5 h	-				0.82	(0.023)	*0.63*	*(0.068)*

^*^ Spearman correlation analysis.

^**^ Kendall correlation analysis.

Non-significant correlations are in italic.

As far as detectable, both IL-31 and IL-13 serum levels did not change 5 and 24 h after nasal allergen challenge compared to baseline (data not shown). IL-31 serum levels were only detectable in the subject who showed a low nasal IL-31 level already 10 min after nasal allergen challenge.

## Discussion

Our study demonstrates for the first time that IL-31 is released into nasal secretions after nasal pollen allergen challenge in patients with allergic rhinitis. The first indication for a role of IL-31 in allergic rhinitis was provided by the characterization of in vitro IL-31 production in pollen antigen–induced PBMC responses in patients with allergic rhinitis [14].

Okano and collegues reported that around two thirds of these PBMCs produced detectable amounts of IL-31 in response to pollen allergens in vitro in association with the severity of allergic rhinitis [14]. We observed nasal IL-31 at detectable levels in five of seven allergic rhinitis patients, four of these peaked at 5 h after nasal provocation. Furthermore, we also found IL-31 contents to show correlation trends to symptoms and thus the severity of allergic rhinitis, suggesting that IL-31 production might lead to a deterioration in the pathophysiology of allergic rhinitis. Due to the limited number of participants with detectable local cytokine levels (and hence some zero values) after nasal allergen challenge we suggest to speak of correlation trends even in case of significant p-values. Our finding that local IL-31 levels did not correlate with itching after a single nasal allergen challenge in allergic rhinitis volunteers contrasts to the previously described link between IL-31 and itching in atopic skin disease [1,10] and may suggest other functions of IL-31 in allergic rhinitis.

Generally, accumulating evidence supports a pathophysiological role of IL-31 in atopic dermatitis and other allergic diseases [1,2,10,11], however its exact mechanisms have not been finally resolved yet [5,8,32,33]. IL-31 may enhance skin inflammation through the induction of several chemokine genes such as CCL17, CCL22 and CCL1 in human keratinocytes [1] which subsequently leads to the recruitment of T-cells, and in turn may become new sources of IL-31 [2]. IL-31 can induce cytokine and chemokine production from human bronchial epithelial cells [9] and eosinophils [34]. Similar pathophysiological mechanisms might also play a pivotal role in the late phase of nasal inflammation of allergic rhinitis, given the influx of inflammatory cells such as eosinophils, basophils, T- and B-cells into the mucosa and potential interactions with resident epithelial cells [35-38].

IL-13 regulates inflammatory and type 2 immune responses [15]. It shares many similarities with IL-4, as both cytokines use IL-4Ralpha as receptor subunit [16,39]. In allergic rhinitis, IL-13 is expressed in the nasal mucosa of patients with perennial allergic rhinitis and after allergen provocation [18,19,40]. In nasal secretions of patients with seasonal allergic rhinitis, IL-13 appeared between 4 and 8 h and peaked 6 h after nasal allergen challenge [19]. We included the type 2 cytokine IL-13 in our study because IL-31 mRNA expression in skin samples of patients with atopic dermatitis or allergic contact dermatitis was reported to be correlated with IL-13 levels [12]. Furthermore, allergen-induced IL-31 protein production by PBMCs in patients with allergic rhinitis was significantly and positively correlated with the production of IL-13 [14]. Our results confirm previous findings describing an involvement of IL-13 in nasal late-phase response in allergic rhinitis [18,19]. Furthermore, our finding of a correlation trend between nasal IL-31 and IL-13 concentrations in the late phase of the allergic reaction following a single nasal allergen challenge may imply that one cytokine induces the other or, alternatively, may point to a common mechanism of induction.

In a mouse model of allergic rhinitis, an essential contribution of IL-13 to the late-phase response in allergic rhinitis was shown by means of either IL-13^−/−^ (knockout) mice or a soluble IL-13 inhibitor [20]. Therapeutical interventions in allergic diseases such as allergen-induced asthma specifically targeting IL-4, IL-5 or IL-13 cytokines caused none to moderate improvement regarding disease severity and symptoms [41]. This suggests a partial redundancy of these cytokines and/or contributions of other mediators to this disease. Indeed, administration of mutant IL-4 protein that inhibits the binding of IL-4 and IL-13 to IL-4R complexes, has recently shown efficacy in the treatment of allergen-induced asthma [42]. If IL-31 is an interesting candidate for therapeutical interventions in allergic rhinitis remains to be elucidated. This could for example be investigated by inhibition of IL-31 via antibodies [10] or specific inhibitors [43].

The limitations of our study such as the small number of volunteers and the relatively small number of timepoints could be overcome in future studies. Nevertheless, more detailed investigations of the timecourse should also include more sensitive IL-31 protein detection methods. Moreover, healthy control subjects undergoing nasal allergen challenge could be included in future studies. Additionally, functional assays to explore the roles of IL-31 on the cellular influx and its effects on resident cells in allergic rhinitis should be considered.

## Conclusions

We demonstrated the release of IL-31 into ipsilateral nasal secretions after unilateral nasal allergen challenge in patients with allergic rhinitis. We found a correlation trend of IL-31 contents with the severity of allergic rhinitis. Furthermore, we confirmed previous reports about the increase of IL-13 in nasal secretions particularly in the late-phase response in allergic rhinitis. The concentrations of this central type 2 cytokine showed a correlation trend to IL-31 following a single nasal allergen challenge. These findings suggest that IL-31 exerts a pathophysiological role in allergic rhinitis.

## Competing interests

The authors declare that they have no competing interests.

## Authors' contributions

Conceived and designed the experiments: RB, MW. Participated in the study co-ordination: RB, MW, JS. Performed nasal provocation tests: MR, IS. Performed the experiments: RB, MR, IS, GAM. Analyzed the data: RB, MW, MR, IS. Contributed reagents/materials/analysis tools: KS, JW, JS. Wrote the draft: RB. Critically revised the manuscript: MW, JW, RB. All authors read and approved the final manuscript.
